# Interleukin 22 and its association with neurodegenerative disease activity

**DOI:** 10.3389/fphar.2022.958022

**Published:** 2022-09-13

**Authors:** Wenjian Chen, Jianpeng Wang, Huaizhi Yang, Yuankai Sun, Bangjie Chen, Yuchen Liu, Yanxun Han, Ming Shan, Junfeng Zhan

**Affiliations:** ^1^ Department of Orthopaedics, Anhui Provincial Children’s Hospital, Hefei, China; ^2^ School of First Clinical Medical College, Anhui Medical University, Hefei, China; ^3^ School of Pharmacy, Anhui Medical University, Hefei, China; ^4^ Department of Oncology, The First Affiliated Hospital of Anhui Medical University, Hefei, China; ^5^ Department of Orthopaedics, The Second Affiliated Hospital of Anhui Medical University, Hefei, China; ^6^ Department of Otolaryngology, Head and Neck Surgery, The First Affiliated Hospital of Anhui Medical University, Anhui Medical University, Hefei, China; ^7^ Department of Neurosurgery, The First Affiliated Hospital of Anhui Medical University, Hefei, China

**Keywords:** neurodegenerative disease, IL-22, central nervous system, neuroinflammatory, immune infiltration, nanotherapy

## Abstract

It is worth noting that neuroinflammation is well recognized as a symptom of neurodegenerative diseases (NDs). The regulation of neuroinflammation becomes an attractive focus for innovative ND treatment technologies. There is evidence that IL-22 is associated with the development and progression of a wide assortment of NDs. For example, IL-22 can activate glial cells, causing them to generate pro-inflammatory cytokines and encourage lymphocyte infiltration in the brain. IL-22 mRNA is highly expressed in Alzheimer’s disease (AD) patients, and a high expression of IL-22 has also been detected in the brains of patients with other NDs. We examine the role of IL-22 in the development and treatment of NDs in this review, and we believe that IL-22 has therapeutic potential in these diseases.

## Introduction

NDs are a leading cause of death and morbidity worldwide, particularly among the elderly. The clinical manifestations and pathological changes vary among patients with NDs. However, they often share certain characteristics such as progressive malfunction and neuronal loss. These shared changes may be associated with abnormal deposition of misfolded proteins in the brains of patients with NDs. These shared changes may be related to the abnormal deposition of misfolded proteins in the brains of patients with NDs, which are inducers of neuroinflammation and glial cell activation (Cai et al., 2014). Glial cells play a key role in the occurrence and development of NDs (Heneka et al., 2015). Activated glial cells can produce and release high doses of toxic substances such as IL-1α, IL-1β, and TNF-α, as well as oxygen-free radicals and nitrous oxide, leading to an inflammatory response that affects synaptic function and kills neurons (Cao et al., 2018).

Immune cells, including a fraction of T helper (Th) cells and innate lymphocytes, generate IL-22, a unique cytokine. In diverse tissues, IL-22 exhibits opposing effects, ranging from pro-inflammatory to protective effects (Sonnenberg et al., 2011; Zenewicz and Flavell, 2011), which are largely depending on the environment. On the one hand, IL-22 can amplify inflammation alone or in combination with other cytokines and cause abnormal epithelial cell proliferation and differentiation. On the other hand, IL-22 induces the expression of pro-survival and anti-apoptotic genes like Bcl-2 and Bcl-xL and the expression of proliferation-related cytokines like c-Myc and cyclin D1. IL-22 also enhanced tissue protection by improving the barrier function. IL-22 has been implicated in the pathophysiology of a variety of organs in several studies (Table 1).

**TABLE 1:** Target tissues and physiological effects of IL-22.

	Tissue	Target cell	Mode of action	References (PMID)
IL-22	Thymus	Thymic epithelial cells (TECs)	·Promotes TEC proliferation and survival	31195136, 21573786, 25246251, and 33278390
			·Endogenous regeneration of thymopoiesis after acute thymic damage	
IL-22	Synovium	Fibroblasts	·Promotes proliferation of fibroblasts	29922283, 32756816, 24056519, and 30619268
			·Induces expression of CCL-2 and RANKL	
IL-22	Liver	Hepatocytes, liver stem cells and progenitor cells	·Enhances regeneration from tissue damage	21465510, 20842630, 22262131, 16212920, 15122762, 17919941, and 21708106
			·Promotes proliferation of liver stem and progenitor cells	
IL-22	Skin	Keratinocytes, dermal cells, and fibroblasts	·Promotes proliferation of keratinocytes and dermal fibroblasts	24378801, 16619290, 14675174, 17083366, 19830738, 17277128, 19330474, 19641206, 24655295, 23664643, 24390134, and 23395647
			·Induces CXCL-1, -2, -5 and -8; defensins; and S100 molecules	
IL-22	Lungs	Bronchial epithelial cells and fibroblasts	·Promotes proliferation of epithelial cells	24942687, 21647421, 18264110, 21297073, 21794904, 21998459, 21789181, 24577508, 24442439, and 24531835
			·Regeneration from tissue damage	
IL-22	Pancreas	Acinar cells, islet α-cells, and β-cells	·High constitutive expression of IL-22R	15122762, 16212920, 14675174, 11798462, and 25408874
			·Induces Reg3β, Reg3γ, Bcl-2, and Bcl-X_L_	
IL-22	Kidney	Tubular epithelial cells	·Promotes tissue regeneration in a TLR4-dependent fashion	11035029, 24459235, 17261606, 21625390, 23659670, and 10875937
			·Polymorphisms of IL-22R lead to nephropathy in children	
IL-22	Gut	Stem and progenitor cells, epithelial cells, and myofibroblasts	·Targets stem and progenitor cells and more mature epithelium	16143135, 22723890, 18978771, 22854709, 18949018, 24991784, 15120652, 19564350, 24239045, 21391996, 21194981, and 18264109
			·Induces S100 proteins; Reg3β, Reg3γ, Bcl-2, and Bcl-X; and defensins	

IL-22 has been linked to a number of chronic autoimmune and inflammatory illnesses, including multiple sclerosis (MS) and psoriasis (Pan et al., 2013). Significantly, higher amounts of IL-22 have also been found in the brains of patients with a range of NDs, and recent research showed that IL-22 generated by leukocytes that invade the brain leads glial cells to activate, promoting disease development. In this review, we will detail the basic properties of IL-22 and its role in the development and treatment of NDs, especially in AD and MS.

## Overview of IL-22

IL-22 is a cytokine belonging to the IL-10 family, which was initially discovered as an IL-10-related T-cell-derived inducer (Sabat, 2010). Like other members of the IL-10 family, the secondary structure of IL-22 is helical (Wolk and Sabat, 2006), and IL-22 in humans consists of 146 amino acids, which is 80.8% similar to IL-22 in mice. IL-22 is derived from a variety of leukocytes, and its expression is regulated by a variety of transcription factors (Pan et al., 2013) (Figure 2).

T lymphocytes were the first cell type to be recognized as an origin of IL-22. Among them, Th1 cells were the first Th cell subset found to produce IL-22 (Wolk et al., 2002). In the existence of IL-12, Th1 cells develop and release the cytokine interferon-γ (IFN-γ). On the other hand, Th17 cells that generate IL-17 and exhibit RORt are considered to be the main source of IL-22 in Th cells (Chung et al., 2006; Zheng et al., 2007). The expression of IL-22 is not linked to IL-17 or RORt expression in human CD4^+^ T cells (Volpe et al., 2008; Volpe et al., 2009). Only 10–18% of IL-22-producing CD4^+^ T cells in human peripheral blood also produce IL-17 (Duhen et al., 2009). Naive T cells develop into IL-17-producing Th17 cells when IL-6, IL-1β, and TGF-β simultaneously stimulate naive T cells, and IL-23 promotes the survival of Th17 cells. Except for TGF-β, all of these cytokines stimulate IL-22 synthesis in T cells (Zheng et al., 2007). In mouse Th17 cells, TGF-β suppresses IL-22 synthesis while increasing IL-17 synthesis (Zheng et al., 2007; Rutz et al., 2011). The foregoing clarifies why various Th17 cells are classified as IL-17-only Th17 cells or IL-17/IL-22 Th17 cells. In addition, there is a Th cell that produces only IL-22 without co-expressing IL-17 or IFN-γ. These cells are defined as Th22 cells (Duhen et al., 2009; Eyerich et al., 2009; Trifari et al., 2009). They do not express T-β and have undetectable low levels of RORt (Duhen et al., 2009). Both Th22 cells and Th17 cells require IL-6 stimulation for activation. IL-6 and TNF-β have a synergistic effect in the activation and promotion of IL-22 secretion of Th22 cells, whereas vitamin D boosts IL-22 production even more. Clones derived from Th22 cells were shown to have rather consistent cytokine profiles across a variety of polarization regimes (Eyerich et al., 2009). However, adding extra IL-1β caused the creation of IL-17 and IL-22. On the other side, TGF-β increases the synthesis of IL-17 while effectively inhibiting the production of IL-22 in Th22 cells (Duhen et al., 2009).

The IL-22 receptor (IL-22R) is a member of the cytokine receptor class II family. It is constituted by the IL-22 receptor complex, which includes IL-22R1 and IL-10R2 (also known as IL10RB). IL-22R1 expression may be seen in non-immune organs such as the skin, lungs, and kidney, as well as, greatest notably, the pancreas (Wolk et al., 2004). However, IL-10R2 is widely expressed in lymphocytes (such as T, B, and NK cells) (Wolk et al., 2004). In recent studies, researchers discovered that IL-22R is expressed in the brain tissue. The IL-22-binding protein (IL-22BP), which is produced by a separate gene and contains the ectodomain of class II cytokine receptors, is a kind of soluble IL-22 receptor. It has been demonstrated to inhibit cellular binding and signal transduction of IL-22 and modify IL-22 bioavailability *in vitro* (Xu et al., 2001; Nikoopour et al., 2015). IL-22 and IL-22BP have a greater affinity than IL-22 and IL-22R1 (Andoh et al., 2005). The low dissociation rate of the IL-22/IL-22BP complex indicates that it is relatively stable. The fact that IL-22 is the only IL-10 family member with additional binding proteins underscores the need to fine-tune its activity. IL-22BP may partly assist the stability and systemic spread of IL-22, in addition to limiting its local effects (Andoh et al., 2005).

## IL-22 in neurodegenerative disease

### IL-22 in Alzheimer’s disease

AD is an ND and the most prevalent cause of dementia. Up to now, about 50 million people worldwide suffer from dementia due to aging populations, and the number of people with dementia is projected to reach 152 million by mid-century, with the largest increase in low- and middle-income countries (Qiu et al., 2009). Over the past few decades, community surveys in Japan and China have found a marked increase in the prevalence of AD (Chan et al., 2013; Ohara et al., 2017). In particular, women have higher morbidity and mortality than men (Collaborators G. B. D. D., 2019). Additionally, from 2000 to 2018, the number of patients dying from AD in the United States increased by 146.2%, making AD the fifth leading cause of death among older adults in the United States. The pathological hallmarks of AD are neurofibrillary tangles and amyloid-β peptide (Aβ) deposition in the brain. Aβ is a major cause of neuronal death in vulnerable areas including the neocortex and hippocampus, which may contribute to behavioral and functional abnormalities in AD (Gandy and Greengard, 1994). The buildup of Aβ in the brain, according to the amyloid hypothesis, is the key factor underlying the pathogenesis of AD. Tau-containing neurofibrillary tangles arise when extracellular Aβ production and clearance are out of equilibrium. However, the amyloid cascade theory cannot completely explain the pathophysiology of AD (Hardy and Selkoe, 2002). Later studies on AD pathogenesis have extensively recognized the close functional relationship between the immune system and the central nervous system (CNS). Microglia are the most significant immune cells in the neural milieu in the CNS, and they may be stimulated to phagocytose and remove Aβ, as well as enhance the production of inflammatory cytokines to speed up a neuronal injury (Hong et al., 2016). These findings suggest that immune system dysfunction is linked to the onset of AD. Additionally, in AD, peripheral immune processes interact with the CNS. Monocytes, macrophages, neutrophils, and T cells from the peripheral blood may infiltrate the brain and play a major role in the genesis of AD (Polfliet et al., 2001; Ziegler-Heitbrock, 2007; Baik et al., 2014; Gate et al., 2020). Recent research found that high levels of activated CD4^+^ and CD8^+^ T lymphocytes in the peripheral blood were highly linked to cognitive impairment and magnetic resonance imaging (MRI) abnormalities in particular brain areas in AD patients (Lueg et al., 2015). The etiology of AD is unknown, but it may be linked to the activation of cytokine production in the brain by Aβ since research found that peripheral T cells activated with Aβ *in vitro* secreted more pro-inflammatory cytokines (Monsonego et al., 2006).

Prior research found that AD patients had no change in the total number of CD4^+^ and CD8^+^ cells but an increased percentage of circulating immune cells, particularly lymphocytes that generate IL-17, IL-6, IL-22, and IFN-γ (Gouya et al., 2014). In AD patients, interleukins (IL-21 and IL-22, etc.) generated by Th17 cells, as well as transcription factors (RORγ, etc.) involved in Th17 cell growth, are significantly increased (Saresella et al., 2011). Although further research is needed on the role and regulatory mechanism of Th17 cells in AD occurrence and development, current research practices reveal that their functions are related to released cytokines, such as Th17 cell infiltration in AD patients’ brains induced b**y** β-42 injection, and in an AD rat model, levels of IL-17 and IL-22 were found to be higher in the brain parenchyma, cerebrospinal fluid, and serum (Zhang et al., 2013). According to Kebir et al. (2007), IL-22 seems to be involved in the breakdown of the blood–brain barrier (BBB). IL-17R and IL-22R were increased in AD patients’ BBB endothelial cells, according to Vitaliti et al. (2019). When IL-22 and IL-17 were co-cultured with human brain endothelial cells, occludin and zonula occludens (ZO)-1 levels were found to be dramatically decreased; this result was also confirmed in experimental autoimmune encephalomyelitis (EAE) mice. Nevertheless, the specific mechanism through which IL-22 causes BBB permeability is unknown. In addition, immune cells that penetrate the brain, such as Th17 cells, may create cytokines that bind to receptors on neurons, resulting in neuronal death and NDs (Tzartos et al., 2011).

### IL-22 in multiple sclerosis

**FIGURE 1:**
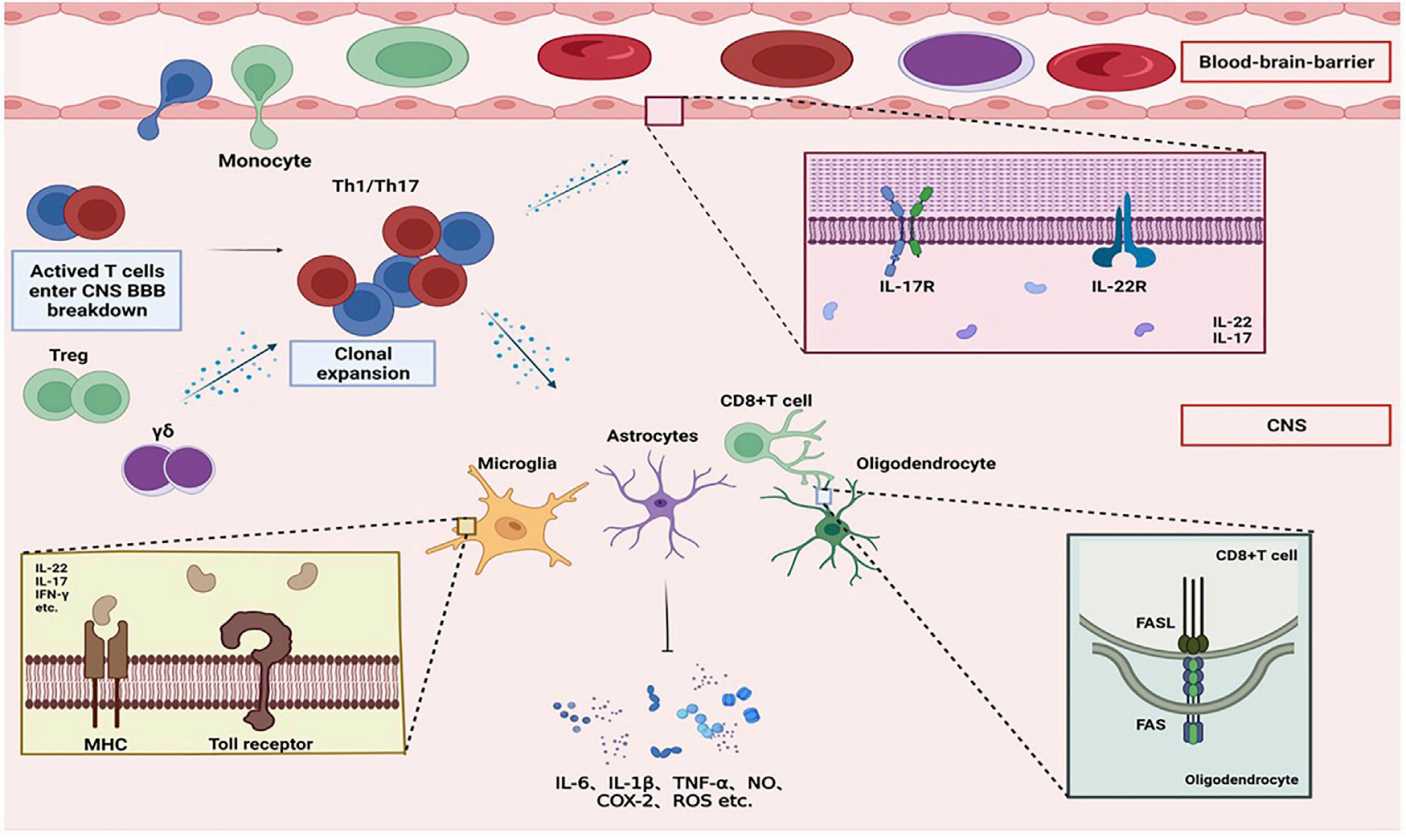
Overview of molecular mimicry. Mononuclear macrophages in the body phagocytose and digest antigenic substances (such as erroneous protein deposits in neurodegenerative diseases) and transmit antigenic signals that have common antigenicity with myelin basic protein to helper T cells. After activation, it enters the CNS, activates effector T cells, releases a large number of cytokines, and activates complement and B cells, resulting in the activation of microglia and astrocytes and the release of neurotoxic substances, oligodendrocyte apoptosis, and myeloid cells. The sheath is damaged, eventually leading to the onset of neurodegenerative diseases.

MS is by far the most prevalent chronic inflammatory disease of the CNS in the world, impacting over 2.5 million individuals globally. According to a systematic analysis of MS (Collaborators G. B. D. M. S., 2019), in 2016, an estimated 2,221,188 people worldwide were living with MS, equivalent to a prevalence of 30·1 cases per 100,000 people. From 1990 to 2016, the age-standardized prevalence rate was estimated to have increased by 22·47 cases per 100,000 people. Age-standardized prevalence rates are higher than 120 per 100,000 in North America and some Nordic countries, moderate (60–120 per 100,000) in some countries in Europe and Oceania, and lowest (<60 per 100,000) in northern Africa and the Middle East, Latin America, Asia, Oceania, the Caribbean, and Sub-Saharan Africa. Notably, the global prevalence of MS varies by gender. Among prepubertal children, the prevalence of MS is similar in boys and girls. During adolescence, the curve begins to diverge, with the prevalence increasing more in girls than in boys. This pattern continued until the end of the sixth decade of life when the sex ratio was 2:1 in favor of women. Among older adults, prevalence rates generally continue to climb in women but decline slowly in men. Although the exact etiology of MS is uncertain, it is assumed to be an autoimmune disease caused by autoreactive lymphocytes reacting to CNS autoantigens (Dendrou et al., 2015). MS has a wide range of clinical symptoms, including sensory and visual difficulties, tiredness, cognitive abnormalities, and motor dysfunction (Compston and Coles, 2008). BBB damage, infiltration of macrophages, T cells, and B cells into the CNS, and local activation of microglia and astrocytes all contribute to inflammation, demyelination, gliosis, neurodegeneration, and primary axonal damage in MS (Frischer et al., 2009; Dendrou et al., 2015). This process can be explained by the “molecular mimicry” theory (Figure 1).

T cells, B cells, and activated macrophages/microglia are the main immune cells in the immune infiltrating brain of MS patients (Pittock and Lucchinetti, 2007). Different CD4^+^ T cell subsets all play essential roles in MS immunopathogenesis. Th1 and Th17 cells play an important role in the initiation and progression of MS. IL-17, IL-6, IL-21, IL-22, IL-23, and TNF-α are inflammatory cytokines generated by Th17 cells, of which IL-17A is the most important cytokine in Th17-mediated encephalopathy. However, new research has shown that IL-17A has only a minor impact on the onset and course of MS. In contrast, the importance of IL-22 in MS advancement has increased significantly. IL-23 is crucial for the development of autoimmune diseases. IL-23 can encourage Th cells to generate IL-17. However, a new study has shown that the IL-22 gene is the most highly expressed gene in Th cells after IL-23 stimulation. IL-22 receptor (IL-22R) is a heterodimer composed of IL-10 receptor-2 (IL-10R2) and IL-22R1, and its expression site is similar to that of the IL-17A receptor (IL-17RA), which mainly exists on the stromal cells of the CNS, such as endothelial cells, epithelial cells, and IL-17RA (Weiss et al., 2004; Boniface et al., 2005; Bettelli et al., 2007). Because of the apparent close relationship between IL-22 and IL-17, including that their receptors are expressed by similar cell types, IL-22 may have a synergistic role with IL-17 in the development of CNS disease. IL-22 also affects barrier surface integrity in a variety of clinical diseases, and when combined with IL-17, IL-22 appears to disrupt the integrity of the BBB (Sonnenberg et al., 2010). This procedure has been demonstrated in the brains of AD patients. The mechanism by which they alter BBB permeability in MS patients may be similar to that in AD patients’ brains. Lymphocytes penetrate the CNS *via* a breach in the BBB, and the infiltrating lymphocytes secrete numerous cytokines that exacerbate disease progression (Kebir et al., 2007). To further explore the relationship between IL-22 and MS, Katharina K et al. (Kreymborg et al., 2007) induced EAE using IL22^−/−^ mice, which were surprisingly fully sensitive to EAE compared to controls. Further study found that these EAE mice had significantly increased the expression levels of IL-22 in the brain and spinal cord. The finding in human patients is also consistent with the results of animal experiments, with MS patients having elevated IL-22 levels in the plasma, white matter, and MS plaques compared with normal controls (Perriard et al., 2015). During EAE onset (Vellios and van der Zee, 2020), IL-22 levels increased markedly at the peak of the disease and dropped sharply during recovery, while IL-10 and IL-17 levels remained unchanged (Almolda et al., 2011). These findings suggest that IL-22 may have a more important role in the development of MS than IL-10 and IL-17.

Another key subset of CD4^+^ T cells, Tregs, is impaired in MS patients. Tregs are one of the most critical cells in autoimmune illnesses, and they may play a role in both controlling and preventing disease progression. Despite the fact that the amount of Tregs in MS is unaffected, their function is diminished, which may contribute to disease development and progression (Huan et al., 2005). CD4^+^CD25^+^FOXP3^+^ Tregs have been reported to inhibit effector T-cell activation and proliferation. The expression of the cellular marker CD25 and the transcription factor forkhead box P3 (FOXP3) characterizes them. Compared with the healthy control group, the FOXP3 mRNA level in MS patients was decreased, and further detection showed that the proportion of CD4^+^CD25^+^FOXP3^+^ Tregs in the total peripheral blood mononuclear cells (PBMC) of the patients was decreased. Jin Z et al. (Zhen et al., 2017) suggested that IL-22 is involved in inhibiting the function of Tregs cells in MS patients, thereby indirectly promoting the activation and proliferation of effector T cells and promoting the development of the disease. When Jin Z et al. stimulated CD4^+^ T cells from MS patients with IL-22, the expression of FOXP3 was significantly downregulated.

However, the mechanism of action of IL-22 in the pathogenesis of MS is not fully understood. Existing studies suggest that IL-22 may play a pathogenic role by regulating the activity of glial cells. In EAE-induced animal brain tissue, IL-22R expression increased with disease progression, and high expression levels of IL-22R were found in microglia, astrocytes, and oligodendrocytes.

In active MS lesions, “classically activated” microglia are assumed to be important for myelin phagocytosis, antigen presentation to T cells, and the production of pro-inflammatory cytokines (Lassmann et al., 2012). In a mouse model of EAE, microglial paralysis had been found to postpone the development of EAE and lower clinical severity (Heppner et al., 2005). However, whereas IL-22 activation of microglia in the brain has been extensively described in other ND models, the effects of IL-22 on microglia in MS have not been explored. Microglia as a therapeutic target for MS has been a hot topic in recent years, and thoroughly understanding the control of IL-22 on the activity of microglial cells in MS patients might aid with targeted treatment.

Traditionally, astrocytes are thought to respond only when neurons are demyelinated and formed glial scars (Brosnan and Raine, 2013). However, recent research has shown that astrocytes are important players in the early stages of MS (Farina et al., 2007; Sofroniew, 2015; Ponath et al., 2017). Astrocytes perform several functions in the progression of MS, including recruiting lymphocytes (Farina et al., 2007; Ransohoff and Brown, 2012; Liddelow and Hoyer, 2016), encouraging tissue damage (Bleasel and Frost, 1986; Warren et al., 1992; Pitt et al., 2000; Smith et al., 2000; Nourbakhsh et al., 2021), limiting inflammation, and promoting lesion healing (Sofroniew, 2015; Tschoe et al., 2020). In recent years, it has also been found that astrocytes in the brain also support B-cell survival and have been implicated in CNS-compartmentalized inflammation and progressive CNS damage (Touil et al., 2018). IL-22R is strongly expressed on astrocytes in the brains of MS patients, particularly in MS plaques or surrounding blood vessels. IL-22 has been found to protect astrocytes in an inflammatory environment in a study as by Perriard et al. (2015). They found that primary human astrocytes treated with IL-22 had a better survival rate. It is possible that anti-apoptotic pathways are involved (Ponath et al., 2018).

The development of MS is accompanied by oligodendrocyte (OL) dysfunction and death, which leads to demyelination and neurodegeneration that exacerbate MS. Furthermore, increased expression of Fas on the glial membrane surface in MS lesions has been observed (Dowling et al., 1996; Hoftberger et al., 2004). FasL on CD8^+^ T cells increased Fas on glial cell interaction, leading to the apoptosis of glial cells (Hovelmeyer et al., 2005).

### IL-22 mediates neuroinflammation in neurodegenerative diseases

Most NDs, including AD, Parkinson’s disease (PD), MS, and amyotrophic lateral sclerosis (ALS), are related to neuroinflammatory processes (Lucin and Wyss-Coray, 2009), for example, highly insoluble Aβ peptide deposits and neurofibrillary tangles constitute a unique trigger for inflammation in the brains of AD patients (Akiyama et al., 2000; Gonzalez et al., 2014). Inflammatory mediators also increase the processing of amyloid precursor protein (APP) in multiple ways, generating a vicious cycle of AD development (Heneka et al., 2010). This adds to the evidence that IL-22 has a function in NDs.

IL-22 is a powerful pro-inflammatory cytokine. Inflammatory cytokines including COX-2, PGE2, IL-6, and TNF-α have a role in various degenerative brain illnesses, according to pathological research (Ennerfelt and Lukens, 2020; Shahdadi Sardou et al., 2020; Daily et al., 2021). The expression levels of inflammatory cytokines were upregulated in IL-22-treated neuronal cells and glial cells. IL-22R is constitutively expressed in BV2 mouse microglia and HT22 hippocampal neurons, and the same results were obtained in mouse brain tissue, particularly its expression in the hippocampus and cerebellum with increased inflammation. Although microglia have protective activity, hyperactivated microglia release high levels of inflammatory cytokines such as IL-6, IL-1β, and TNF-α, leading to neuronal death and chronic inflammation, which is the degenerative main cause of encephalopathy (Cervellati et al., 2020; Wu et al., 2021). In addition, the expression level of COX-2 is quickly upregulated in inflammatory circumstances, and IL-22-treated BV2 and HT22 cells revealed a considerable rise in COX-2 mRNA expression (Lee et al., 2022), and PGE2 synthesis in both cells enhanced (Nikoopour et al., 2015). This shows that by activating COX-2, IL-22 may boost PGE2 synthesis. Using the Affymetrix GeneChip Mouse Gene 2.0 ST array, Dahae Lee et al. (2022) evaluated the expression levels of inflammatory cytokine genes in HT22 cells, following IL-22 administration and discovered that roughly one-third of inflammatory cytokines were upregulated.

### Regulatory mechanism of IL-22 in neurodegenerative diseases

A lot of research has been conducted on NDs in recent years. However, in-depth research into its molecular processes is still lacking. It is worth mentioning that the involvement of the JAK/STAT signaling pathway in NDs has been steadily uncovered, and it is safe to say that the JAK/STAT signaling pathway plays a key role in ND pathogenesis (Trager et al., 2013; Yan et al., 2018; Jain et al., 2021).

The JAK/STAT signaling pathway is a signal transduction pathway stimulated by cytokines involving the activation of two families of proteins discovered and cloned in the early 1990s: the Janus kinase (JAK), which comprises four tyrosine kinases (JAK1, JAK2, JAK3, and TYK2), and the signal transducer and activator of transcription (STAT) contains seven structurally and functionally related proteins (STAT1, STAT2, STAT3, STAT4, STAT5A, STAT5B, and STAT6). In the CNS, the aberrant JAK/STAT signaling pathway is associated with hormone secretion, neuroinflammation, and tumorigenesis, which will lead to CNS dysregulation. Dysregulation of the CNS mainly affects the state of neurons/glial cells. Nevado-Holgado et al. (2019) studied AD patients, demonstrated that the JAK/STAT signaling pathway has a key role in the development and progression of AD, and pointed out some potential AD therapeutic targets in this signaling pathway. Reichenbach et al. (2019) studied STAT3-deficient APP/PS1 mice and found that STAT3-mediated astrogliosis is an important therapeutic target for AD. In addition, in the EAE animal model, the role of the JAK/STAT signaling pathway was also confirmed. In the EAE animal model, STAT4 regulates IFN-γ production by autoreactive Th1 cells. IFN-γ can promote macrophage STAT1 expression and stimulate macrophage M1 differentiation. Th17 cells also generate GM-CSF in the CNS, which promotes macrophage pro-inflammatory polarization through the JAK2/STAT5 signaling pathway (Andersson et al., 2015).

**FIGURE 2:**
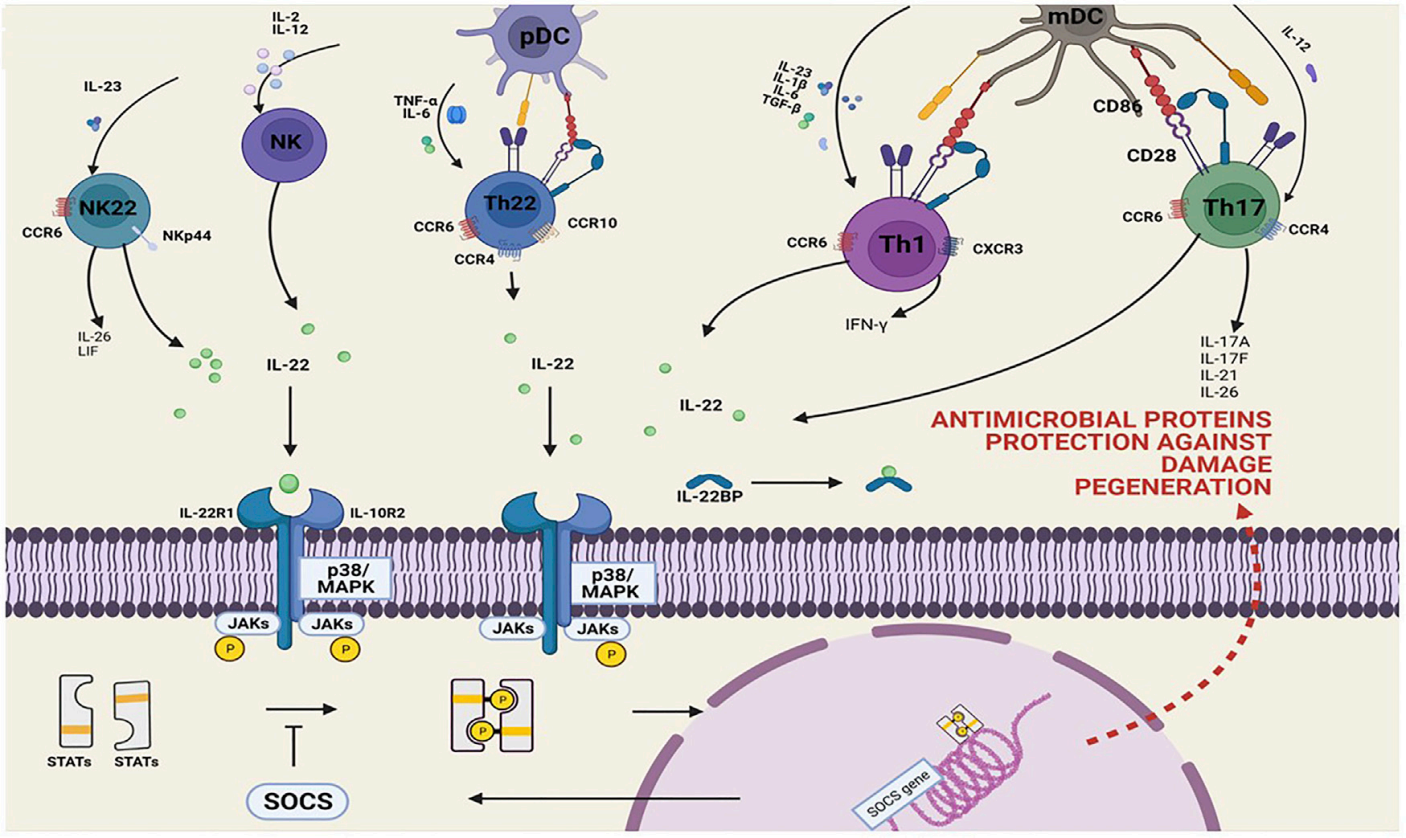
Overview of the production and action signaling pathways of IL-22. IL-22 is often, but not always, correlated with IL-17 expression. Subpopulations of CD4^+^ T cells, CD8^+^ T cells, innate T cells, and innate lymphoid cells are sources of IL-22, IL-22, and IL-17 or IL-17. The main cytokines driving IL-22 expression are IL-23, IL-6, and IL-1β. Certain cell types require IL-12, IL-18, IL-21, and TNF-α to produce IL-22. TGF-β inhibits IL-22 production in all T-cell subsets but induces IL-17 in these cells; IL-22 signals through its cognate receptor/JAK complex, resulting in downstream phosphorylation of STAT (homo- or heterodimer). Translocation of these STAT complexes to the nucleus drives transcription of genes involved in processes ranging from inflammation to angiogenesis and survival. The control of activity of this pathway involves different mechanisms including regulation of the phosphorylation state of JAK and STAT by phosphatases, or of the JAK kinase activity by SOCS (suppressor of cytokine signaling), for example.

Although the JAK/STAT signaling pathway has been shown to be activated in NDs, the underlying mechanisms remain unknown. The relationship between IL-22 and the JAK/STAT signaling pathway is well known (Figure 2). JAKs (JAK1 and Tyk2) are used in IL-22 signaling to transmit downstream phosphorylation signals such as mitogen-activated protein kinase (MAPK) signaling (ERK1/2, MEK1/2, JNK, and p38 kinase), signal transducers, and transcription activators (STAT1, STAT3, and STAT5) (Lejeune et al., 2002). When IL-22 binds to its receptor complex, receptor-associated JAK1/Tyk2 kinases are activated, causing phosphorylation of downstream proteins. Meng et al. described the function of Src homologue-2, consisting of protein tyrosine phosphatase 2 (Shp2), in the IL-22 signaling pathway. Studies had shown that IL-22 induced IL-22R1 phosphorylation, and Shp2 bound to tyrosine-phosphorylated IL-22R1 upon IL-22 stimulation. Furthermore, Tyr251 and Tyr301 of IL-22R1 were required for Shp2 binding to IL-22R1. The binding of Shp2 to IL-22R1 and activation of Shp2 protein tyrosine phosphatase were critical for MAP kinase activity, signal transduction, and IL-22-activated STAT3 transcriptional phosphorylation. These research studies showed that Shp2 plays a crucial role in the IL-22-mediated signal transduction pathway (Meng et al., 2010).

In addition to the JAK/STAT signaling pathway, the binding of IL-22 to cellular receptors also promotes the activation of MAPK and the p38 signaling pathway (Andoh et al., 2005; Ikeuchi et al., 2005; Wolk et al., 2006; Liu et al., 2012; Mitra et al., 2012). However, these IL-22-activated MAPK and p38 signaling pathways are both cross-linked with the JAK/STAT signaling pathway. The NF-κB signaling pathway is also involved in the development of NDs, with increased cell surface FAS expression in IL-22-treated CG4 cells and mouse oligodendrocytes. Compared with the controls, IL-22 greatly enhanced the phosphorylation of MSK-1 and then activated p65, a key protein in the NF-κB signaling pathway, demonstrating that IL-22 activated the MSK-1/NF-κB signaling pathway and enhanced the Fas expression on the surface of glial cells (Zhen et al., 2017). In NDs, all of the aforementioned signaling pathways influence disease progression (Jiang et al., 2009; Jia et al., 2011; Noubade et al., 2011; Arumugam et al., 2012; Tagoe and Putterman, 2012). Therefore, manipulating IL-22 and its signaling pathway might be a viable treatment method for NDs (Pan et al., 2013).

### IL-22 as a clinical therapeutic target potential

Disease-modifying therapy (DMT) has been widely used in recent decades as an effective disease-modifying therapy to treat NDs (Gross and Corboy, 2019; Cole et al., 2021), and there are now two approved drugs used to treat AD, namely, cholinesterase inhibitors and N-methyl-D-aspartate (NMDA) antagonists. However, cholinesterase inhibitors and NMDA antagonists are only effective for treating AD symptoms but do not completely cure or prevent AD. In addition, the enormous limitations to the vast majority of drugs (98% of small-molecule drugs and 100% of large-molecule drugs) in crossing the BBB further increase the therapeutic difficulty of treating NDs. Stem cell therapy, also known as regenerative therapy, uses stem cells or their derivatives to improve the repair response of dysfunctional and damaged tissue. The goal of stem cell therapy is often focused on cell replacement or providing environmental enrichment. Stem cell therapy has revolutionized medicine over the years, and its therapeutic applications provide a valuable and attractive option for the treatment of a variety of diseases, including NDs (Sakthiswary and Raymond, 2012). However, there are some inevitable adverse consequences to this strategy. As a result, developing better therapies is critical. Immunotherapy has received increased attention in recent years, and excellent therapeutic benefits have been established in the clinical treatment of a variety of disorders. IL-22 has promise as a possible target for treating AD, MS, and other NDs due to its crucial involvement in the inflammatory and proliferative cascades. As previously observed, IL-22BP has an inhibitory influence on the function of IL-22 and has been identified as a risk gene for MS (Beyeen et al., 2010). We think that IL-22 neutralizers are prospective medications not only just for MS but also for other NDs since soluble cytokine receptors have been demonstrated to be useful as a treatment for inflammatory diseases (Moreland, 1999). Wang et al. (2012) found that the expression levels of inflammatory cytokines (TNF-α and IL-6) were considerably lowered in the mouse brain using the IL-22^−/−^ mouse model. TNF-α and IL-6, as previously indicated, aid in the recruitment and maintenance of inflammatory Th17 cells in the tissue (Kebir et al., 2007) and may contribute to disease development and progression (Paganelli et al., 2002; Ng et al., 2018). Wang et al. (2017) injected Aβ1-42 into the mouse hippocampus to create an Alzheimer’s animal model, and they found that the model mice had a higher level of IL-22 in the hippocampus and cortex. After IL-22 was highly expressed in the mouse brains, the mice’s disease improved significantly. According to the findings, IL-22 may be a possible therapeutic target for NDs, and this is a promising therapeutic strategy.

Hu et al. (2019)analyzed cerebrospinal fluid samples from AD, MS, PD, and DLB patients and found that four cytokines (IL-10, IP-10, IL-8, and TNF-α) related to innate immunity and subtypes of helper T cells (Th1, Th2, and Th17) have much greater levels of expression in the cerebral fluid of patients with degenerative disorders than healthy people. Rodrguez-Sáinz Model C et al. performed a cross-sectional investigation of 17 randomly chosen patients during an MS episode, measuring TNF-α and IL-10 expression levels in the blood and cerebrospinal fluid (CSF) of the patients. The results showed that TNF-α and IL-10 expression levels in the blood and CSF of MS patients were greater than in healthy controls. The high levels of TNF-α and IL-10 (in the serum and cerebrospinal fluid) that accompany the onset of NDs indicate simultaneous expression of cytokines Th1, Th2, and Th17, and given the relationship of IL-22 to these three cells, we suspect IL-22 also has untapped clinical potential in patients with NDs.

## Future outlook

Nanotherapy is a new treatment method with lower toxicity and fewer side effects that has become more popular in recent years. Therapeutics and theranostics based on nanoparticles (NPs) have been developed for a variety of ailments, particularly for the treatment of different kinds of malignancies (Muntimadugu et al., 2017; Gong et al., 2019). However, crossing the BBB with a carrier for small-molecule therapies is particularly challenging because the BBB significantly restricts the use of nanotherapeutics in CNS illnesses like NDs. Finding effective delivery systems to help drugs cross the BBB and successfully localize and bind to targets is a challenge in the field of treatment of NDs.

Lohan et al. (2017) employed carbon nanotubes as a carrier for the medication BRB, effectively delivering neuro drugs to brain microglia and slowing the course of AD. BRB has several limitations, including low bioavailability, poor intestinal absorption, and limited CNS penetration, despite its great therapeutic benefits. Conti et al. (2017) developed a functional liposome that removed Aβ42 and delayed disease progression. NLCs offer excellent biodegradability, physical durability, low toxicity, and superior degradability, but they have drawbacks such as gelation, lipid compartment development, and fundamentally poor integration rates, among others. These issues obstruct their use in clinical practice (Naseri et al., 2015). Li et al. (2021) recently developed poly (lactic-co-glycolic acid) (PLGA)-based NPs. The researchers discovered that the nanoparticles’ surface coating interacted with the BBB and nerve cells in many ways to improve drug absorption and that PS 80-coated NPs (PS 80-NPs) loaded with tau siRNA effectively suppressed tau expression in mouse nerve cells. In addition, inorganic nanoparticles, such as magnetic nanoparticles (Cui et al., 2018), metal-rich nanoparticles (Naziroglu et al., 2017), and quantum nanoparticles (Gupta et al., 2010), are also promising options for treating NDs.

Despite the fact that there have been several studies published on the use of nanocarriers to cure and regulate NDs, no relevant clinical trials have been reported. The use of simple, repeatable, and low-cost nanomaterial preparation methods is a key barrier to scaling up these materials. The protein corona effect, the impact of cell sex, type, and passage number on cell therapy, and the need for patient-specific cells are all difficulties that must be addressed in nanocarrier clinical trials. The majority of the scholarly literature on nanomedicine ignores these difficulties (Mahmoudi, 2018). Overall, there are still numerous issues in the clinical translation of nanocarriers to be resolved. The creation of vectors that target IL-22 silencing has considerable advantages for ND remission, and it may be a major approach for ND therapy.

## Conclusion

A variety of cytokines are related to the occurrence and development of NDs, among which IL-22 is a key factor in the process of NDs. Although the mechanism of IL-22 in NDs is yet unknown, a growing body of evidence from *in vivo* and *in vitro* models suggests that IL-22 might be a feasible therapeutic target for NDs. However, it should be emphasized that the majority of the data on IL-22 came from animal models, which may or may not be relevant to people (or other experimental ones). Furthermore, the function of IL-22 in prevalent NDs such as PD and ALS has not been investigated. As a result, further research is required, particularly in human systems, to fully comprehend the function of IL-22 in NDs.

## Abbreviations

NDs, neurodegenerative diseases; TNF-α, tumor necrosis factor-α; AD, Alzheimer’s disease; MS, multiple sclerosis; IFN-γ, interferon-γ; TGF-β, transforming growth factor-β; Aβ, amyloid-β peptides; CNS, central nervous system; EAE, experimental autoimmune encephalomyelitis; Tregs, regulatory T cells; OL, oligodendrocyte; PD, Parkinson’s disease; ALS, amyotrophic lateral sclerosis; APP, amyloid precursor protein; STAT, signal transducer and activator of transcription; JAK, Janus kinase; MAPK, mitogen-activated protein kinase; DMT, disease-modifying treatment; CSF, cerebrospinal fluid; NPs, nanoparticles; BRB, berberine; NLCs, nanostructured lipid carriers; MRI, magnetic resonance imaging.
